# Progress in Cancer

**Published:** 1903-04-11

**Authors:** 


### Progress in Cancer.

Henry Morris[1] regards cancer at first as a local disease and on this view enumerates four groups of inoperable cases : (1) Primary cancer affecting inaccessible parts and organs ; (2) primary cancer which, though originating in an accessible part or organ of the body, has been allowed to extend beyond the limits within which an operation is prudent and complete removal possible ; (3) certain cases of acute diffuse carcinoma and very rapidly-growing or widely-infiltrating new growths of exceptionally virulent character ; (4) recurrent cancer, where the disease has recurred in multiple metastatic foci, or in parts beyond the limits of removal. Operations for cancer should be more divided into the two classes, (1) those employed with a reasonable hope of eliminating the disease, and (2) those whose object lies in giving relief from pain, prolonging life, restoring function, and other palliative procedure. He questions the advisability of extending the operations for mammary cancer to the lengths adopted by many in the present day ; or of wide excisions in cancer of the pharynx, larynx, œsophagus, or stomach ; or of those operations for cancer of the uterus where, to be radical, more than the uterus must be removed. In Kraske's excision of the rectum the gut should not be bound down by adhesions and the connective tissue infiltrated, for in such cases operation leaves frequently a worse condition than would otherwise occur.

In cancer of the cervix, the whole organ should be removed per vaginam ; but in gastro-enterostomy it is doubtful, where even successful, whether there is a real gain to the patient. Operations might be more frequently undertaken for excision of the urinary bladder, and great relief is experienced in partial operations on the tongue, removal of sterno-mastoid where the triangles of the neck are extensively involved, and in amputation of the arm in some cases of mammary cancer leading to painful swelling, etc., of that limb, or division of the brachial plexus. More might be done to remove the ills of sepsis ensuing upon fungating growths of rectum and cervix. Reviewing the results in the serum and Coley's fluid treatment of cancer Morris concludes that there is no evidence of value in carcinoma, while in spindle-celled sarcoma alone is Coley's fluid of proved value, and even then less than half the cases are attended by disappearance of the tumours ; and the treatment is far from being free from danger to life from collapse, septic absorption, from too rapid necrosis of the tumour, from pyæmia and erysipelas, while the attending unpleasant symptoms, such as high temperature, rigors, nausea, etc., are great drawbacks. Cancroin Vlaieff's serum have yielded no hopeful results. Webb's soap solution and other substances injected do not recommend themselves.

With regard to Beatson's oöphorectomy, he says, "I doubt whether it will take a permanent place in surgery ; still its present position seems to be this—that surgeons may justifiably recommend a trial of it in cases of chronic inoperable mammary carcinoma, after fully explaining the nature of the operation, the uncertainty of the result, and the doubtful prospects of permanence should a cure be obtained.' He advises that the oöphorectomy be rather held in reserve for possible recurrences than performed at the same time as the removal of the breast. He has noticed no good derived from thyroid feeding in cancer. In Finsen's light, and in the *x*-rays, we have, he says, the most successful treatment for rodent ulcer, as far as is known as yet. This is true, not only of cases otherwise inoperable, but also of operable cases because of their excellent cosmetic results, and of their effect upon insidious and non-evident foci. There are, nevertheless, cases of rodent ulcer which resist the light and others which resist the *x*-ray treatment, and some of these cases are successfully treated by excision and caustics.

Sarcoma and epithelioma, with other forms of carcinoma, are best treated, whenever possible, by early excision, and all forms of treatment hitherto tried in inoperable cancers of these kinds are uncertain and inconstant in their effects and unreliable as to the durability of the results they produce. In the vast majority of cases they are quite without palliative influence of any kind, except, possibly, in relieving pain. The boundary line between what are considered operable and inoperable cases needs revision from time to time; that the tendency to extend the limits of operable cases needs in some instances to be restricted, and in others there may prove room for further extension. Investigations into both the cause and nature of cancer are of the first importance as being more likely to ultimately lead to cure than any treatment at present known. With few exceptions the attempts to cure cancer by means other than early and free operations have been hitherto almost invariably futile.

Double oöphorectomy was performed for recurrent mammary carcinoma in a woman aged 42 by McGavin.[2] The patient had not reached the climacteric and was therefore a case favourable for the operation. In spite of this, however, recurrent nodules again appeared about six months later and no evidence of any advantage from the operation has appeared. No note is made of thyroid extract having been used nor, according to Alexis Thomson,[3] is this adjuvant treatment to be considered essential. The latter also draws the conclusion from collected tables that there is no age at which the operation is contraindicated, for marked improvement has been noticed in patients who have passed the menopause. In McGavin's case decrease in the size of the axillary tumour was noticed, but attributed to a decrease in the blood supply consequent upon the operation for removal of the breast. Thomson states that disease of the axillary or supra-clavicular glands, with or without swelling of the arm, yields less rapidly and less frequently after oöphorectomy, but has been observed to disappear or subside in a considerable proportion of cases.

[1] Brit. Med. Jour., Oct. 25. [2] Lancet, Oct. 18. [3] Brit. Med. Jour., Nov. 8.

*(To be continued.)*

